# Human Social Evolution: Self-Domestication or Self-Control?

**DOI:** 10.3389/fpsyg.2020.00134

**Published:** 2020-02-14

**Authors:** Dor Shilton, Mati Breski, Daniel Dor, Eva Jablonka

**Affiliations:** ^1^The Cohn Institute for the History and Philosophy of Science and Ideas, Tel Aviv University, Tel-Aviv, Israel; ^2^The Department of Communication, Tel-Aviv University, Tel-Aviv, Israel; ^3^Centre for Philosophy of Natural and Social Science (CPNSS), London School of Economics, London, United Kingdom

**Keywords:** self-domestication hypothesis, human social evolution, language evolution, music evolution, emotional control

## Abstract

The self-domestication hypothesis suggests that, like mammalian domesticates, humans have gone through a process of selection against aggression – a process that in the case of humans was self-induced. Here, we extend previous proposals and suggest that what underlies human social evolution is selection for socially mediated emotional control and plasticity. In the first part of the paper we highlight general features of human social evolution, which, we argue, is more similar to that of other social mammals than to that of mammalian domesticates and is therefore incompatible with the notion of human self-domestication. In the second part, we discuss the unique aspects of human evolution and propose that emotional control and social motivation in humans evolved during two major, partially overlapping stages. The first stage, which followed the emergence of mimetic communication, the beginnings of musical engagement, and mimesis-related cognition, required socially mediated emotional plasticity and was accompanied by new social emotions. The second stage followed the emergence of language, when individuals began to instruct the imagination of their interlocutors, and to rely even more extensively on emotional plasticity and culturally learned emotional control. This account further illustrates the significant differences between humans and domesticates, thus challenging the notion of human self-domestication.

## Introduction

The notion that humans are “domesticated” far precedes the notion that humans have evolved. Since antiquity, scholars have described humans (in general or in reference to their own particular culture) as domesticated, which generally referred to their “civility”: their distance from a wild or savage state of being. It was common for writings on the subject to be entangled with various value judgments, with some considering the superiority of a domesticated state, while others described it as a kind of physical and mental degeneration. Coupled with the tradition of differentiating human cultures on the basis of the extent to which they were “domesticated,” much literature on the subject promoted views of social hierarchies in civility, which were later used as a pseudo-scientific rationale for racist and eugenic political movements (reviewed in Brüne, 2007). This stain on the intellectual history of human domestication theories illustrates the complex social meanings of the concept, and its consequent ambiguity when used in explaining human evolutionary processes.

It was Darwin who first critically discussed self-domestication from an evolutionary perspective. While he conceded that humans are similar to domesticates in exhibiting extreme phenotypic variability, he nonetheless argued that the term domestication would be misapplied in the case of human evolution:

“It is, nevertheless, an error to speak of man, even if we look only to the conditions to which he has been exposed, as ‘far more domesticated’ […] man differs widely from any strictly domesticated animal; for his breeding has never long been controlled, either by methodical or unconscious selection. No race or body of men has been so completely subjugated by other men, as that certain individuals should be preserved, and thus unconsciously selected, from somehow excelling in utility to their masters.” (Darwin, 1871, pp. 28–29)

That said, Darwin’s study of domesticated species recognized the package of traits that many mammalian domesticated species share, which includes morphological traits such as skeletal changes (shorter muzzle, decreased heart size, reduced teeth size, short and curly tail, floppy ears), physiological traits such as altered and usually more numerous reproductive cycles, and the retention of many juvenile behavioral features. Decades later, Boas (1938) observed that many of these traits were also shared by humans, and suggested that this was due to similar selective pressures. Specifically, Boas suggested that in both cases, traits like de-pigmentation, shortening of the face, and the loss of reproductive periodicity were partially the result of a more protective environment and a diet of softened, processed food. Notably, Boas argued that various social laws and prohibitions (e.g. marriage regulation, prohibition of infanticide) could also have had selective effects, and was thus anticipating concepts like cultural niche construction, which would later prove crucial for understanding human evolution.

In 1959, a still-ongoing experiment on the domestication of silver foxes was initiated by Dmitri Belyaev, Lyudmila Trut and their colleagues in Novosibirsk (Belyaev, 1979; Trut, 1999; Trut et al., 2006; Dugatkin and Trut, 2017). Belyaev’s experimental design has become central to the current formulation of the self-domestication hypothesis. Belyaev defined domesticated behavior as “the ability of animals to have direct contact with man, not to be afraid of man, to obey him, and to reproduce under the conditions created by him” (Belyaev, 1979). The experimenters consequently selected for tameness – the degree to which human contact resulted in docile, rather than aggressive, behavior. Tameness was estimated through limited human contact: a gloved hand was introduced into a cage with a young fox cub, and its reaction was monitored (Trut et al., 2009). Importantly, the procedure did not involve any prolonged contact or training, and selection was based purely on the perceived propensity toward tame behavior. Belyaev thus separated as best he could the genetic component, and created a speeded up evolutionary process. It should be stressed, however, that during typical processes of social evolution, including domestication, selection is much more complex and taming includes many additional factors, including priming and learning processes. In the case of human social evolution, these involve social and cultural interactions within and between groups.

It is also important to note that the original fox population used in Belyaev’s experiments had been bred in captivity for about 50 years before the domestication experiment was initiated, so the farm foxes do not represent a completely wild population (Lord et al., 2019). Most of the foxes were either aggressive, fearful, or aggressively fearful in response to human contact, but a few displayed less aggressive and more exploratory reactions toward the gloved hand (Belyaev, 1979). About 10% of the most tame in each generation were selected (Trut et al., 2009). Several generations later, the experiment had produced a population of foxes whose reaction to human contact was the opposite of that exhibited by most of the original population, with fear and aggression superseded by willful and positive engagement. As predicted by Belyaev, the behavioral changes were accompanied by physiological and morphological changes, as well as by changes in mating habits. The foxes had shortened legs, tails, snouts and upper jaws; floppy ears, curly tails, and altered coat color patterns; mating became more frequent and no longer strictly seasonal; supernumerary and non-essential B chromosomes became more frequent; the pattern of inheritance of a pigmentation pattern (a white star on the forehead) was found to be non-Mendelian. At the hormonal level, which is involved in many behavioral changes, the domesticated population exhibited reduced activity of the Hypothalamus Adrenal Axis (HPA axis), as well as higher levels of serotonin and higher activity of key enzymes related to serotonin synthesis and degradation, both of which appear to be critical for the facilitation of tame behavior. Interestingly, a line of foxes selected for increased emotional reactivity (enhanced fearful-aggressive behavior) also showed some characteristics of the domestication syndrome (white spotting and changes in stress hormones), suggesting that different variations in the regulation of the same developmental pathway may have been under selection in both the tame and the aggressive lines. The fox selection experiments are reviewed in Jablonka and Lamb (1995); Markel and Trut (2011), Dugatkin and Trut (2017), Wilkins (2017, 2019), and Lord et al. (2019).

Discussions of self-domestication since the late-20th century have centered around Belyaev’s definition of domestication – in particular, his emphasis on tameness and reduced aggression rather than adaptation to human-made environments. Later research has put into question the robustness of his definition. Lord et al. (2019) argued that the evidence for a widely shared suite of traits among animal domesticates, a “domestication syndrome” (DS)^1^, is inconclusive: none of the DS traits are shared by all domesticates, although a reduction in brain size, changes in craniofacial characteristics and increased variation in coat color are observed in most. Nevertheless, in general there is a “family resemblance” among domesticated species, which, we believe, renders the notion of DS useful (but see Lord et al., 2019 for a dissenting view).

Coppinger and Coppinger (2001) proposed a different route to domestication from that of Belyaev. They suggested that the domestication of wolves (*Canis lupus*) into dogs involved an initial phase in which less nervous members of the group became better dump-feeders in human habitats, and eventually formed a separate population. During this stage there was “self-domestication,” which involved adapting to feeding opportunities in and near human habitats (becoming a synanthropic species), initially without intentional human intervention.

Hare and colleagues have suggested that bonobos (*Pan paniscus*) have also undergone self-domestication, meaning, more generally, that they went through a process in which selection for reduced aggression led to DS traits (Hare et al., 2012; Hare, 2017). Citing evidence for reduced aggression and physiological and morphological differences between bonobos and chimpanzees (e.g. Rilling et al., 2012), Hare and colleagues proposed a model of bonobo evolution involving the formation of female coalitions, which thwarted male aggression and male alliances. They called the outcome of this process of selection against aggression “self-domestication.” While reduced aggression (seen in less competitive feeding habits and increased social tolerance) is emphasized, other critical behavioral factors are also mentioned. These include more stable parties, extended female sexual receptivity and a much less significant reduction in relative brain size (when compared to species domesticated by humans). This raises the question of whether this complex suite of physiological traits and social behaviors is indeed best described as an outcome of a “self-domestication” process, rather than as the outcome of selection for cooperation and emotional control that is observed in many other highly social mammals. In the case of humans, these questions are particularly pertinent.

Human self-domestication is usually characterized as a process of selection against aggression, and more recently as selection for pro-sociality. For example, Sánchez-Villagra and van Schaik (2019) characterize the human self-domestication hypothesis (HSD) thus: “The current version of the HSD hypothesis postulates that selection for reduced aggressiveness in human evolution led to physiological, psychological, and behavioral changes, specifically to social tolerance (p. 136).” Hare (2017), who recognizes the importance of selection for self-control in human evolution, also emphasizes the similarities of human social evolution to that of domesticates and stresses selection for pro-sociality and against aggression: “The human self-domestication hypothesis (HSD) draws on comparative, developmental, fossil, and neurobiological evidence to show that late human evolution was dominated by selection for intragroup pro-sociality over aggression (p. 157).” The stress on selection against aggression and for docility is also highlighted by Francis (2015), and with qualifications, by Wrangham (2018), who focuses on a reduction in reactive, high arousal, non-calculated aggression.

Hare (2017) underscored the complexity of the changes undergone by humans and suggested that increased self-control is the hallmark of human social and cognitive-affective evolution. We agree with this suggestion, which was based on Hare and Tomasello’s earlier proposal that a reduction in emotional reactivity was the pre-condition for human cognitive evolution (Hare and Tomasello, 2005). In the second part of this paper we extend these suggestions and propose that engagement in music and in linguistic communication contributed significantly to the evolution of cognitive and emotional plasticity in the genus *Homo*. However, because of the differences between humans and domesticates, we take issue with suggestions that human social evolution, especially early evolution, is best described in terms of self-domestication. We suggest that the evolution of unique human characteristics requires an explanatory framework based on emotional and cognitive plasticity, a framework that goes beyond the selection against aggression and for pro-sociality that is described in most characterizations of self-domestication.

Other accounts of HSD stress the similarities in the protective environments of humans and their domesticated species (Thomas, 2013), emphasizing the effects of relaxed selection pressures on both human and domesticate evolution (Brüne, 2007). Our approach differs from these accounts by (1) focusing on earlier hominin evolution, beginning with *Homo erectus*, when most human-specific cooperative and morphological traits seem to have already evolved; (2) suggesting that human social evolution is more similar to the evolution of pro-social behavior in other highly social mammals, which is associated with increased sophistication of social structures and increased cognitive and emotional plasticity; and (3) emphasizing the unique social-cultural selective environment of humans, which, we argue, shaped and amplified our species’ cognitive and affective plasticity. The recent evolution of humans, especially after the split with Neanderthals, is interpreted as the outcome of intense cultural evolution driven by language, musicking and other cultural strategies (Heyes, 2018), rather than by selection against aggression.

## Similarities and Differences Between Humans and Domesticates

The HSD hypothesis is based on the assumption that since humans share several (though not all) traits common to many animal domesticates, they have undergone a similar selection process (Thomas and Kirby, 2018). In addition to morphological and behavioral similarities, there is also some evidence that selection targeted genes in the same developmental pathways, including genes expressed in neural crest cells. Wilkins et al. (2014) suggested that gene mutations leading to slightly reduced expression of genes in the neural crest underlie the DS and can explain why so many traits are shared among domesticates. Neural crest cells are pluripotent embryonic cells, derived from the neural tube in early embryogenesis. The cells migrate and give rise to neuroendocrine cells, pigment cells, neurons and glial cells of the sensory, sympathetic, and parasympathetic nervous systems and many of the skeletal and connective tissue components of the head. Since the structures and processes associated with the neural crest are also related to the DS traits, the hypothesis offers a unifying explanation. Genetic variation in the regulatory genetic networks (GRNs) of these pathways have indeed been shown to characterize several domesticates (Simões-Costa and Bronner, 2015; Theofanopoulou et al., 2017; Wilkins, 2019). Moreover, variation in neural crest genes, as well as variations in genes expressed in cortical regions of the brain (including the neo-cortex) have been observed in neurodevelopmental pathways that affect neural plasticity and learning (see Theofanopoulou et al., 2017 for a comparison focusing on humans, and Wang et al., 2018 for gene expression in silver foxes).

In addition to variations in DNA base sequences, epigenetic variations may also be involved in the DS, since it was shown that the expression of the DNA methyltransferase genes differs between domesticated and control foxes (Herbeck et al., 2017). There is also evidence of significant and multiple epigenetic differences between jungle fowl and domesticated chickens: selection for fearful and non-fearful behavior in the jungle fowl for only five generations led to divergent DNA methylation in 22 genomic regions in hypothalamus cells, some of which were associated with neural functions and cellular metabolic pathways relevant to the stress response (Bélteky et al., 2018). A study of very recent domesticates of sea bass, which show no genetic differences from wild fish, found that these recent domesticates have epimutations (differences in patterns of DNA methylation) in various tissues, with about one fifth of the persistent epimutations being in genes that are expressed in embryonic structures, including the neural crest. Furthermore, the epimutated genes coincide with mutated genes in established domesticates (Anastasiadi and Piferrer, 2019). It is therefore plausible that a comparative study of epigenetic (e.g. methylation) differences among domesticates and humans will reveal many more substantial similarities and differences than gene-sequence differences, but at present there are only a few comparative studies that address this question.

Table 1 presents a comparison between human traits that correspond to traits that are said to characterize the DS in (i) apes (bonobo compared to chimpanzee), (ii) dogs/wolves (feral and domestic dogs compared to the gray wolf), and (iii) foxes selected for tame behavior and wild, unselected ones. A detailed comparison of the traits associated with DS that includes many other species of domesticates is presented and discussed in Sánchez-Villagra et al. (2016).

**TABLE 1:** Comparison between modern humans, apes, and domesticated and non-domesticated canids (dogs/wolves and tame/wild foxes).

Species evolved factors	Modern humans	Bonobo/chimpanzee	Dog/wolf	Domesticated silver foxes/unselected foxes
**Morphology**				
Morphological variability	Highly variable	Both species less variable than humans	Dog breeds highly variable	Tame foxes highly variable
Mean brain volume (cm^3^)	1239.8; increase in human brain size throughout most *Homo* evolution; 10% reduction during the last 10,000 years^1^	345.6/375.1^1^	100.4/139.8^2^	Negligible differences^3^
Cranium	Evolved globularity emerged in *H. sapiens* lineage^4^	Average bonobo endocranium is more rounded and less elongated than that of the chimpanzee^5^	Reduced facial length in dogs compared to wolves^6^	Changes in the tame strain are similar to changes during dog’s early domestication^7^
Sexual dimorphism in body mass (male/female ratio)	1.16^1^	1.35/1.31^1^	Varies with size of dog breed; 1.27 in wolves^8^	1.2 in wild red fox^9^; no available data on experimental groups
Pigmentation	Depigmentation of the sclera is unique to humans^10^	Depigmentation of lips and tail tuffs in bonobos^11^	Depigmentation of coat in dogs^12^	Depigmentation of coat in tame foxes^7^
**Endocrinology**				
Serotonin receptor	Receptor expression in the amygdala’s central and accessory basal nuclei is significantly higher compared than in the chimpanzee and bonobo (*Pan*) genus^13^	Receptor expression in amygdala’s basal nuclei is significantly higher in bonobos^14^	High levels of variation in serotonin receptor and transporter genes of the dog^15^	Higher levels of serotonin and serotonin receptors in the brain of tame foxes^16^
Oxytocin receptor Prolactin	Genetic variation linked with social behavior, empathy and autism^17^; epigenetic changes in oxytocin receptor gene associated with autism and unemotional traits^18^ Adult male prolactin levels rise in response to infant cries during fatherhood and during participation in sexual acts^23^	Fixed genetic variation in both species compared with the polymorphisms found in humans; five additional genetic polymorphisms found in chimpanzees but not in bonobos or humans; their functional importance has not been determined^19^ Prolactin levels in male chimpanzees spike throughout sexual development^24^; no available data on bonobos	Genetic variation in dogs related to differences in social behavior^20^, was not identified in wolves^21^; epigenetic differences among dogs associated with differences in appeasing behavior^22^ Prolactin levels rise in all wolf pack members during pup-rearing period^25^, but are not correlated with paternal behavior in male dogs^26^	No available data No available data
Cortisol	Cortisol levels are sensitive to environmental conditions and are socially regulated during postnatal development^27^	Cortisol levels in bonobos, but not in chimpanzees, change during competition over food and show a greater increase in response to social stressors^28^	Baseline cortisol levels in wolves depend on dominance hierarchies^29^, whereas in dogs they are sensitive to human caregivers’ personality and lifestyle^30^	Reduced cortisol levels in all tame strains; highly reduced in pregnancy and lactation^31^
Testosterone levels in males	Increase during out-group competition; decrease during in-group competition, pair-bonding and co-sleeping with child^23^	In male chimpanzees but not bonobos, there is pubertal and adulthood increases and level-changes during competition over food^32^	Increased testosterone in wolves is seasonal and tied to reproduction, whereas most dog breeds continuously maintain elevated levels ^33^	Lower levels of plasma testosterone in tame foxes^34^
**Variation in DNA and in gene expression**	Humans show many differences when compared to apes; there are also differences between anatomically modern humans and archaic humans; archaic humans do not show adaptive sweeps in genes related to DS characteristics^35^	The observed divergence of neural and social traits in chimpanzees and bonobos has not been associated with differences in protein patterns^36^	Overlap among 15 genes that show adaptive sweeps in both modern humans and dogs (but not in wolves). Of these 4 genes show characteristics associated with the DS^35^	150 genes show different patterns of expression in lines of foxes selected for aggression and tameness; allele frequencies at 176 gene loci, including genes associated with neural crest functioning, are different between the aggressive and tame lines^37^
**Emotional reactivity**				
Aggression	Compared to other primates, humans show high propensity for proactive aggression and low propensity for reactive aggression^38^	Both proactive and reactive aggression in chimpanzees; reduced proactive aggression and reduced severity of reactive aggression in bonobos^39^	Both species show only rare and weak aggression among conspecifics^40^	Tame foxes are very docile and non-aggressive compared to control group^41^
Cooperativeness (pro-sociality)	Early onset of cooperative and pro-social behavior^42^	Cooperation in chimpanzees is limited, and restricted to same-sex pairings whereas bonobos show broader cooperation^43^	Compared to wolves, dogs find it difficult to cooperate with conspecifics ^44^	Tame foxes are more interested in interacting with humans than are wild foxes^41^
Emotional control	Humans can either inhibit, modulate or mobilize aggressive and other emotional responses, depending on ecological conditions, norms etc. ^45^	Bonobos are more socially tolerant than chimpanzees^46^	Dogs show a higher level of inhibitory control than wolves with regard to humans, and can better suppress their immediate drives in favor of delayed rewards^47^	Compared to wild foxes, tame foxes show an increase in exploratory behavior with age, coupled with a substantial decrease in cortisol levels^48^
**Life History**				
Neotenous features	Observed across various anatomical traits of adult humans^49^; gene expression indicates neural neoteny in brain areas involved with social and cognitive skills^50^	Bonobos have pedomorphic cranium, white tail-tufts that characterize juvenile chimpanzees, and play between adults is similar to adult-juvenile chimpanzee play^51^	Dog breeds are underdeveloped to varying degrees with regard to physical and behavioral traits compared to wolves^52^	Tame foxes show a trend for faster sexual maturation accompanied by retarded development of some somatic traits^7^
Length of female reproductive cycle (years)	3.05^53^	4.8/5.2–6.6^54^	0.45/0.7^55^	0.5–1/1^7^
Length of juvenile period (years)	13.3^56^	12/7.2^56^	Juvenile period is similar in both species, but wolves’ sexual maturity may depend on growth in size and on territoriality^57^	Sexual maturation in tame foxes occurs a month earlier on average^7^
**Social behavior and cognition**				
Developmental timing	Long childhood; human brains show an extreme level of postpartum development, followed by an extended period for synaptic pruning that lasts until the mid-20’s^58^	Extended development and maternal-attachment in bonobo infants, with delayed development of social behavior and cognition relative to chimpanzees^59^	The period of socialization in domestic dogs is longer than that observed in wild or socialized wolves^60^	Sensitive period for social development in tame foxes is extended from 45 days to 12 weeks or longer^41^
Reproductive regulation	Mating and child rearing are regulated by cultural group norms^61^; concealed copulations occur regardless of male dominance and status^62^	Reproduction is determined by dominance hierarchies in chimpanzees^54^, whereas in bonobos male reproductive success is influenced by mother’s social status^63^	Reproduction in dogs is controlled by humans; in wolves, the dominant pair breeds while other females are reproductively suppressed, unless food is abundant^64^	Reproduction of tame foxes controlled by humans; in ancestral wild species, female reproduction depends on population density, food supply, and social status^65^
Paternal care	Variable across-cultures and associated with local ecologies and social environments^66^	Similar patterns in both *Pan* species, but bonobo males engage in more playful activity with infants, including sociosexual play^67^	Male wolves provide babysitting and play with infants, whereas provisioning by male dogs is rare and limited^68^	Males in wild populations defend and provision pups^69^; no available data on experimental groups
Alloparenting	Modern humans in hunter-gatherer groups and other social organizations practice alloparenting^61^	Bonobos show more allomaternal care than chimpanzees^70^	Helpers in wolf packs attend to, and provide for pups^64^; provisioning by non-maternal female dogs is rare^57^	Females act as helpers in wild populations^71^; no available data on experimental groups
Infanticide	Relatively rare in hunter-gatherer groups and usually initiated by the mother, when resources are limited or the infant is deformed^61^	Male bonobos assault, but do not attempt to kill, weaned offspring; male chimpanzees commit infanticide^72^	Major mechanism used by dominant feral dog females to suppress reproduction of subordinates; dominant female wolves aggressively prevent copulation by subordinates^73^	Not reported for experimental groups; in farm conditions, infanticide by vixens is correlated with more tense and insecure behavior^74^
Communication and information sharing	Polymodal and variable communication; extensive information sharing and early manifestation of communicative intents and skills^75^	Compared to chimpanzees, bonobos are more sensitive to human gaze direction, use indexical cues in the vegetation when foraging in small groups, and acquire better linguistic skills in experimental settings^76^	Wolves have better skills with regard to gaze following and imitation vis á vis conspecifics, but only dogs gaze at human faces for assistance^77^; both follow human pointing but it appears earlier in dogs than in wolves^78^	Tame pups more skilled in responding to human communicative gestures; novel displays of tail wagging, submissive posturing and barking in adult tame foxes^79^
Play	Advanced pretend play parallels language development^80^; social and pretend play in hunter-gatherers are used to counteract tendencies toward dominance^81^	During juvenile period play-fighting becomes longer and more cooperative in bonobos, whereas in chimpanzees it is more competitive^82^	Juvenile play behavior is maintained in adult dogs^83^	Play during adulthood is more common in tame foxes^84^
				

*^1^MacLeod et al., 2003; Robson and Wood, 2008; Bednarik, 2014; ^2^Comparison taken from dog breed and wolf with similar body masses; see Smith et al., 2018; the variability between different strains should, however, be noted; Lord et al., 2019; ^3^Trut et al., 1991 (quoted in Wilkins et al., 2014); ^4^Neubauer et al., 2018; ^5^Durrleman et al., 2012; ^6^Franciscus et al., 2013 (quoted in Cieri et al., 2014); ^7^Trut et al., 2004; ^8^According to Frynta et al., 2012, Sexual size dimorphism in large breeds is comparable to SSD of wolf; becomes smaller with decreasing body size (see also Moehlman and Hofer, 1997); ^9^Voigt, 1987; ^10^Tomasello et al., 2007; ^11^Kano, 1992; ^12^Coppinger and Coppinger, 2001; ^13^Lew et al., 2019; ^14^Stimpson et al., 2016; ^15^van den Berg et al., 2005; ^16^Popova et al., 1991; ^17^Wu et al., 2005, 2012; Tost et al., 2010; ^18^Kumsta et al., 2013; ^19^Staes et al., 2014; ^20^Kis et al., 2014; ^21^Oliva et al., 2016; Bence et al., 2017; ^22^Cimarelli et al., 2017; ^23^Gray et al., 2017; ^24^Kondo et al., 2000; ^25^Kreeger et al., 1991; Asa, 1997; ^26^Corrada et al., 2003; ^27^Gunnar and Donzella, 2002; Flinn et al., 2011; ^28^Wobber et al., 2010a; ^29^Sands and Creel, 2004; ^30^Schöberl et al., 2017; ^31^Trut et al., 2009; ^32^Wobber et al., 2010a, 2013; ^33^Asa, 1997; ^34^Osadchuk and Shurkalova, 1992; ^35^Theofanopoulou et al., 2017; ^36^Staes et al., 2019; ^37^Wang et al., 2018; ^38^Wrangham, 2018; ^39^Surbeck et al., 2011; Furuichi, 2011; ^40^Range et al., 2015; ^41^Trut, 1999; ^42^Tomasello, 2009; ^43^Surbeck et al., 2017; ^44^Feddersen-Petersen, 2007; ^45^Hare, 2007; Jablonka et al., 2012; ^46^Tan and Hare, 2017; Hare et al., 2007; ^47^Gácsi et al., 2009; Marshall-Pescini et al., 2015; ^48^Trut, 2001; ^49^Skulachev et al., 2017; ^50^Bufill et al., 2011; ^51^Wrangham, 2002; Palagi, 2006; Lieberman et al., 2007; ^52^Udell et al., 2010; ^53^Key, 2000; ^54^Gruber and Clay, 2016; ^55^Jöchle, 1997; ^56^Jones et al., 2009; ^57^Lord et al., 2013; ^58^Zollikofer and Ponce de León, 2010; Casey, 2015; ^59^de Lathouwers and Van Elsacker, 2006; Wobber et al., 2010b; ^60^Coppinger and Coppinger, 2001; Gácsi et al., 2009; ^61^Hrdy, 2009; ^62^Ben-Mocha et al., 2018; ^63^Surbeck et al., 2019; ^64^Montgomery et al., 2018; ^65^Macdonald, 1980; ^66^Fernandez-Duque et al., 2009; ^67^Enomoto, 1990; ^68^Kleiman and Malcolm, 1981; Pal, 2005; ^69^Macdonald, 1979; ^70^Kano, 1992; Furuichi, 2011; ^71^Moehlman and Hofer, 1997; ^72^Gottfried et al., 2019; ^73^Corbett, 1988; ^74^Braastad, 1987; Braastad and Bakken, 1993; ^75^Tomasello, 2008; ^76^Savage-Rumbaugh et al., 1996; Gillespie-Lynch et al., 2014; MacLean and Hare, 2015; ^77^Range and Virányi, 2013, 2014; ^78^Gácsi et al., 2009; ^79^Trut, 1999; Hare et al., 2005; ^80^Lewis et al., 2000; Hughes, 2010; ^81^Gray, 2009; ^82^Palagi, 2006; ^83^Goodwin et al., 1997; ^84^Trut, 2001; Trut et al., 2004, 2009.*

The table shows some similarities between humans, bonobos, dogs and tame silver foxes that conform to the characteristics of the DS. The levels of behavior-affecting hormones, most notably elevated levels of serotonin and oxytocin, which are correlated with reduced emotional reactivity, are increased in humans, dogs and tame foxes, with tame and wild foxes showing clear differences with regard to the genes involved in these pathways (Wang et al., 2018). Another similarity is the juvenilization of morphology, the increase in morphological variation, and the prolonged play period in humans and domesticates. These similarities are thought to reflect parallel evolution affecting the same set of genetic regulatory networks in humans and domesticates, in particular, though not exclusively, in genes controlling developmental networks in which neural crest cells are involved. The data, however, are far from conclusive. When genes showing adaptive sweeps in modern humans (742 human genes) and domesticates (dog, cat, horse, taurine cattle; 691 genes in total) were compared, 41 were shown to be shared by both humans and one or more domesticated species (15 of the 41 were shared with the dog), and only 5 of the 41 were shared between humans and several domesticates. Of these 5 genes, 4 showed variations related to neural, behavioral and morphological characteristics related to the DS and to neural crest pathways. Two genes seem particularly important: BRAF, which affects learning and neural plasticity, and GRIK3, which affects both learning and cranial characteristics (Theofanopoulou et al., 2017). However, as Theofanopoulou et al. (2017) point out, the human data are based on somewhat contested compilations of human genes showing adaptive sweeps. There are also important data limitations that complicate interpretation: identification of adaptive sweeps uncovers selective changes only in protein coding genes, so the regulatory non-coding sequences, which are probably of the greatest significance in the evolution of the relevant regulatory networks, cannot be detected. This means both that the number of overlaps is likely to be underestimated, and that the overlaps identified may not be specific to the DS.

We do not want to downplay the similarities between humans and domesticated foxes and dogs, nor do we question the involvement of neural crest mutations in the DS. We believe, however, that these commonalities can be explained in a way that is not committed to the HSD hypothesis. The neural crest pathways affect such a large suite of morphological, physiological and neural phenotypes that we expect that variations in them will be targeted by social selection whenever there is strong selection for altered emotional reactivity, mate selection, and social cognition – that is in *several* social selective contexts. These include selection for domestic, tame characteristics; selection reducing stress-related behaviors involving the flight (fear) and fight (aggression) responses seen in small island populations of newly introduced animals; sexual selection and social selection for pro-sociality in social mammal and bird groups; and social-cultural selection for human cognition and affect. Sexual selection and changes in diet and climate can also be underlain by variations in developmental processes that involve the neural crest, which lead to cranial modifications such as those seen in Neanderthals and Denisovans.

The table clearly shows that as well as similarities there are also striking differences between humans and domesticates. One important trait that humans do not share with most (80%) domesticates is reduced brain size – in fact, the opposite evolutionary trend is often considered as a hallmark of human evolution. While reduced cranial capacity is in line with other pedomorphic traits of domesticates, an increase in hominin brain size relative to body size has been correlated with changes in diet resulting in higher energy intake, increased technical intelligence, and greater social complexity (Dunbar, 1998; Barton, 2012; DeCasien et al., 2017). The retention of juvenile traits in humans is associated with increased, rather than decreased, brain size because the extended human juvenile period involves a prolongation of neural growth and development (Gould, 1996). As noted by Spurway (1955) in her seminal paper on domestication, the reduced brain size of domesticates can be explained as the result of selection for the breakdown of social structures. To encourage increased growth and reproduction in domesticates, humans selected for the slackening of mating criteria, a shorter period of parental care, reproduction at earlier ages, unresponsiveness to group hierarchy, less discrimination in the choice of food, less territorial defense, and so on. These traits are often associated with diminished perceptual acuity and lead to a social structure that is impoverished relative to that of their wild ancestors, and that is not self-sustaining in the absence of human provisioning (Avital and Jablonka, 2000).

There is evidence for a reduction in endocranial volume in humans in the past 40,000 years and especially the last 10,000 years (Bednarik, 2014), and it has been suggested that this points to selection for pro-sociality. Alternatively, the reduction may be related to the decrease in overall size, to increased sedentism, more reliable food availability and greater safety (Hare, 2017; Thomas and Kirby, 2018). It is, however, important to note that most morphological and behavioral traits that are associated with the DS in anatomically modern humans (e.g. increased social cooperation, neoteny, changes in cranial morphology, reduced sexual dimorphism) are shared by archaic humans, and so preceded the period in which HSD is supposed to have occurred.

There are certainly differences in human morphology as well as in genes when Neanderthals, Denisovans and anatomically modern humans are compared (Hare, 2017), and some changes are in genes affecting pathways in which neural crest cells are involved and that lead to changes in the cranium. In a recent study, Zanlella et al. (2019) showed that there were changes in the chromatin remodeler BAZ1B in neural crest stem cells during the evolution of anatomically modern humans. They found that large-effect mutations in the regulatory region of this gene lead to DS-like cranial and neural disease-related variation in modern humans. More subtle genetic variations in the regulatory regions in this gene differ between modern humans, Neanderthals and Denisovans and may be related to the cranial differences among them. The reduction in average brow ridge projection and shortening of the upper facial skeleton from the Middle Pleistocene to recent times has been linked by Cieri et al. (2014) to a reduction in aggression and increased social tolerance. However, the context in which these cranial and behavioral changes were selected is not clear, and it has not been established that they are the result of selection against aggression rather than, for example, the result of sexual selection, or changes in diet or climate. Finally, the data showing adaptive sweeps in modern humans but not in Neanderthals are very limited (Theofanopoulou et al., 2017). Nevertheless, it is possible that, as Sánchez-Villagra et al. (2016) have suggested, once humans had adopted a more sedentary life style, about 15,000 years ago, there was selection for decreased vigilance similar to that observed in animals that migrate to small islands devoid of predators, which often leads to reduced brain size. This may partially account for the recent reduction in human brain size.

A second important difference between humans and most other domesticates is the types of aggression they display. While humans can be docile and patient with one another in some situations, they can also be extraordinarily violent at others. Wrangham (2018) distinguished between reactive and proactive aggression in order to clarify this apparent oddity. Humans, he suggests, share with chimpanzees a high propensity for proactive aggression (purposeful, target-consistent, low arousal), and share with bonobos a low propensity for reactive aggression (responsive, target-inconsistent, high arousal). However, if self-domestication is defined as selection against reactive aggression, many social mammals, including meerkats and mole rats, should be included in the self-domestication category. Furthermore, when violence occurs, reactive and proactive aggression are often mixed (Allen and Anderson, 2017). Although the decision-mechanisms initiating proactive violence are claimed to be neurally distinct (Blair, 2016), levels of arousal may change during the act itself – a “coldly” premeditated act of violence can be carried out in a state of high arousal. The lower rates of within-group violence among humans compared to other great apes (Wrangham, 2018) may in part be a result of violence being better controlled, both emotionally and socially, rather than the propensity for reactive aggression being simply reduced.

A third crucial difference between humans and domesticates relates to the absence, in the case of humans, of subordination to another species, and an increased dependence on other group members with regards to foraging, hunting and alloparenting. Consider the differences in social ecology between wolves and dogs: dogs feed primarily on human waste, whereas wolves rely mostly on group hunting; dog pups are raised mostly by their mothers (and, in the case of pet dogs, by humans as well), while wolf pups are raised by the entire pack (Marshall-Pescini et al., 2017a). Recent experiments have clarified the impact of the different social ecologies on behavior, showing, for example, that wolves have greater pro-social tendencies toward pack members than do dogs (Dale et al., 2019), that they cooperate better with conspecifics than dogs (Marshall-Pescini et al., 2017b), and that although wolves and dogs are both capable of cooperating with familiar humans, dogs tend to take on more submissive roles (Range et al., 2019). Like wolves, throughout much of their evolutionary history humans relied on group-coordinated hunting and participated in alloparenting. Until the onset of agriculture, they did not rely on living alongside and being provisioned by another species, but rather on their intra-group pro-social tendencies, which allowed them to cooperate with one another. In other words, humans’ social ecology did not require docility toward a domesticator, but rather emotional plasticity that can lead to condition-dependent pro-social behavior among group members, as well as highly aggressive behavior, mainly toward individuals belonging to other social groups. In many ways, human social evolution is more similar to that of wolves than to that of dogs.^2^

Finally, unlike animal domesticates and bonobos, humans can create cumulative cultures (Mesoudi, 2011; Laland, 2017). The cultural learning involved depends on enhanced attention to the actions of others, and this may explain the depigmentation of the sclera in humans, which Tomasello et al. (2007) suggested had evolved to facilitate gaze-following. Uniquely human forms of communication, engagement, and material technologies point to a cognitive and emotional profile that goes well beyond the reduced aggression shown in bonobos. The increased emotional plasticity of humans allows the modulation of emotional reactions on the basis of social situations and expectations: a norm-sensitive emotional control.

We believe that incorporating selection for emotional control and plasticity can better account for human behavior, affect and cognition, than selection for reduced aggression or pro-sociality alone. It can also explain why some traits are shared with domesticates, and others are not. The HPA axis, which affects fear and flight reactions in all vertebrates, is also involved in learning and memory (Sandi and Pinelo-Nava, 2007), so it is likely that mutations and epimutations in this system, and even more so in its regulation by higher cortical regions (which are involved in executive control) will be found in social mammals including bonobos and humans.

Selection for emotional control could account for the continued increase rather than decrease in brain size for most of human evolution. A study of self-control in 36 species of mammals and birds found higher levels of control to be best predicted by absolute brain volume, while also being correlated with dietary breadth in primates (MacLean et al., 2014). There are several brain regions (subcortical, cortical and neocortical) implicated in emotional control. These include the cerebellum, which is more broadly involved with attentional control and social skill-sets Schmahmann (2019), and prefrontal cortical regions that interact with the anterior cingulate cortex to form the executive attention network, which is critical for supporting the development of emotional regulation (Posner and Fan, 2008). Braunstein et al. (2017) pointed to four control systems that have been strongly implicated in implicit and explicit regulation of the emotions: the dorso-lateral prefrontal cortex (dlPFC), which is involved in subjective awareness, cognitive appraisal and strategic control (Lapate, 2018); the ventrolateral prefrontal cortex (vlPFC), which is implicated in the selection of goals; and the dorsal anterior cingulate cortex (dACC) and dorsal medial prefrontal cortex (dmPFC), which are involved in monitoring the compatibility or conflict between intended and actual behavioral outcomes and one’s emotional states. In addition, the posterior parietal cortex (PPC) interacts with the dlPFC, exerts top–down, volitional control over attention and working memory processes, and supports perspective taking and spatial processing. Importantly, the PPC is strongly recruited during reappraisal that involves emotional distancing, suggesting that it regulates perceptions of an emotional stimulus’ relevance or proximity (Silvers and Moreira, 2019). A recent phylogenetic analysis has found that disproportional increases in the volumes of the neocortex and the cerebellum occurred, respectively, at the origins of haplorrhines and of the apes, and not predominantly during the rapid and directional brain evolution observed in hominins. However, the general increase in brain size in humans means that these emotion-related brain regions are nevertheless larger than expected for a primate of similar body mass (Miller et al., 2019). We therefore suggest that the genetic and epigenetic networks underlying the development of these neocortical regions were major targets of selection during human social evolution, and that reduced aggression might be a symptom of broader social plasticity and more nuanced social emotions.

Advocates of HSD may argue that selection for emotional control and plasticity is not entirely distinct from selection against reactive aggression. However, the former is expected to be fundamental to the evolution of social motivation in many highly social animals and does not necessarily lead to a decrease in overall aggression; it is expected to lead to increased aggression in some social contexts and to increased cooperative behaviors in others – patterns of behavior than are seen in wolves and social mongooses. Since selection for emotional control is likely to involve both the early and late developmental pathways that underlie neural development, and since changes in the early pathways have multiple pleiotropic effects, it is to be expected that the behavioral, social and morphological evolution of social vertebrates will be affected by selection for changes in these pathways. Mutations affecting neural crest cells are therefore expected to be associated with several different aspects of social evolution, not just with domestication. We therefore do not find the notion of human self-domestication useful, and believe that the partial analogy with domesticates focuses too much on the reduction of reactive aggression and too little on social organization. With respect to cooperation, selection for emotional control in hominins was essential for alloparental care, cooperative hunting and foraging, and the improvement of lithic technologies, all of which had advantages that compensated for the higher metabolic costs involved with the increase in brain size and connectivity that is required for improved emotional and executive control. With respect to aggression, selection for emotional control better explains the extraordinary range of human violence: humans are far less impulsive than other apes, can better control their aggression in some social conditions, and are able to amplify their aggression in other conditions, leading to extreme cruelty. As we argue below the social-cognitive emotional profile of humans, whose underlying developmental pathways lead to the emergence of traits that partially overlap those that characterize the DS, is the consequence of selection for greatly enhanced emotional control and plasticity, which were linked with the culture-guided evolution of human capacities.

## Human Social Evolution

### Social Emotions and Emotional Plasticity in Pre-linguistic Humans

Early human evolution was marked by three novel and increasingly important behaviors: the production of stone tools (Laland, 2017), the consumption of meat and marrow (Ferraro et al., 2013; Thompson et al., 2019), and, somewhat later, the emergence of alloparenting (Hrdy, 2009). All bear an interesting relation to emotional control, pro-sociality and communication.

The use of sharp-edged stones for flesh removal and marrow extraction is found as early as 3.4 Mya (McPherron et al., 2010). Lithic traditions increased in complexity over time, demanding that individuals not only have the ability to comprehend long, hierarchical sequences, but also have the patience and tenacity to work through them (Pargeter et al., 2019). A knapper attempting to produce a complex tool (e.g. an Acheulian biface) has to keep various sub-goals constantly in mind, and to decide the manner in which he should proceed on the basis of the result of each flake removal. Both emotional and executive control are therefore necessary for the production of a complex stone tool (Stout et al., 2015). As for the social transmission of the skills and knowledge involved, ethnographic and experimental evidence both suggest that it requires flexible and creative mimetic communication and a high degree of pro-social motivation (Shilton, 2019). Experts and novices need to spend plenty of time together, to share a common goal of successful tool production, and to use their gestural communication for the purpose of teaching (Laland, 2017). Through joint knapping interactions, novices learn to see the core as the expert does, and become aware of the various visual cues that guide the next striking action (e.g. striking platforms, step fractures and grain quality). In other words, experts and novices need to establish a common ground based on communicative signals, which many researchers consider to be the starting point of human-specific communication (Tomasello, 2008). The benefits of better stone tools would therefore have promoted emotional control and plasticity, both for patient tool production, and to facilitate the kind of cooperative interactions skill transmission required.

Hunting and foraging skills also became increasingly more advanced during human evolution and, like tool-making skills, relied on cooperative activity and social learning. Even the more conservative scholars in the hunting vs. scavenging debate agree that by 1.5–1.0 Mya hunting was a regular component of hominin subsistence (Domínguez-Rodrigo and Pickering, 2017). The regular consumption of highly nutritious meat, fat and marrow answered the metabolic demands of larger brains. Since brain size is hypothesized to be related to self-control (MacLean et al., 2014), and since such control would improve the motor learning and social learning abilities of hominins (which, in turn, require even more self-control), a positive feedback loop might have been initiated at some point in human evolutionary history (see also Hare, 2017).

A large item of prey that was consumed by many individuals required communicating about it, moving it, guarding it, gathering around it, and eating it together without too many squabbles. It has been suggested that the hunting of megafauna, evident since approximately 1.7 Mya, indicates a concurrent and mutually reinforcing increase in group size and increased cooperative practices (Domínguez-Rodrigo and Pickering, 2017). The nature of plant consumption is more difficult to ascertain archaeologically, but studies in the ∼800,000 years old Acheulian site of Gesher Benot Ya’aqov provide evidence for the consumption of diverse plant species, mainly USOs (underground storage organs) and nuts. The extraction and preparation of these require complex procedures (Melamed et al., 2016) and, as in the case of tool-making, hunting and foraging skills, they were executed and socially transmitted through collaborative efforts in which visual cues in the environment needed to be mutually identified and responded to. USOs, for example, sometimes leave just small traces above ground, and digging implements are needed to retrieve the deeper ones (Thomas, 2006). Tracking, which is essential for hunting, involves recognizing spoors to infer the prey’s location and physical state (Liebenberg, 2013). Selection for these skills involved selection for the emotional disposition and communicative abilities that they require.

Emotional control and pro-sociality were also likely to have been substantially influenced by alloparenting – the care of young by individuals other than their mother. Extensive alloparenting is universal in human societies (Sear and Mace, 2008), and among the great apes unique to humans (Hrdy, 2009). This practice has a proven impact on several other factors distinguishing human evolution and psychology, such as intersubjective abilities, proactive pro-sociality, brain size and altriciality (Hrdy, 2009, 2016; Isler and van Schaik, 2012; Burkart et al., 2014). Alloparenting may have emerged quite early in the hominin line because (i) cooperative breeding is especially likely to evolve in ecologically unstable environments (Hrdy, 2016); (ii) Australopithecus females were estimated to have given birth to babies who were more than 5% of their adult body mass compared to 3% in chimpanzees and 6% in modern humans (DeSilva, 2011); and (iii) there is evidence for extended altriciality in *Homo erectus* (Cofran and DeSilva, 2015). Strong trust relationships have to be formed in order for mothers to allow others access to their young: chimpanzee mothers, for example, are highly protective. Alloparenting may have developed in ecological conditions that kept mothers in close proximity to their familiar and trusted matrilineal kin. Allowing males and less related kin to provision and provide care is indicative of very high levels of group trust and tolerance.

The impact of alloparenting on human psychology is far-reaching, both for caregivers and infants. Fathers show increased oxytocin and decreased testosterone levels compared to non-fathers (Rilling and Mascaro, 2017), and caregivers’ parenting behavior is correlated with distinct brain activation patterns, including circuitries that support, among other things, emotional empathy, comprehension of others’ intentions and feelings, reward and motivation, and anxiety (Glasper et al., 2019). These appear to result in structural changes to the brain during parenting, such as an increase in both mothers and fathers in gray matter volume in the hypothalamus, amygdala and striatum (Kim et al., 2014; Kim, 2016). The prolonged brain maturation of human infants means a prolonged influence of postnatal environmental and social interactions on neural connectivity (Sakai et al., 2011; Miller et al., 2012). Compared to chimpanzees, human infants also show a more rapid increase in white matter volume in the prefrontal cortex, a difference that is probably related to social interactions (Sakai et al., 2011). Hrdy (2016) contrasts this with the much slower maturation of other brain areas, especially those related to motor coordination and mobility, and suggests that it can be partially explained by the greater importance for human infants of assessing the intentions and commitment levels of caregivers and of soliciting care.

All of the cooperative behaviors we have described both increased the adaptive value of emotional control and contributed to the extended pro-sociality of hominins. A life-style based on toolmaking, hunting, foraging, and alloparenting meant that early hominins were uniquely codependent and other-regarding compared to other great apes. The growing importance of cooperative alliances demanded a greater sensitivity to the expectations of others, which led to the emergence of social emotions like embarrassment, shame, guilt and pride, all signaled by the uniquely human blush (Crozier, 2006). The emergence of most of these self-evaluating emotions in development is thought to occur in the second phase of emotion regulation, during which children in their third to sixth year of life become capable of an intrapersonal regulation of their emotional actions and reflections (Holodynski and Friedlmeier, 2006).

### Mimesis, Musical Engagement and Emotional Control

The method most likely to accommodate hominins’ new cooperative behaviors is mimetic communication, or mimesis. Described initially by Donald (1991), the tool-kit of mimesis includes manual and bodily gestures (including the all-important gesture of pointing), facial expressions and vocalizations, mimicking, pantomime, and early musicking. The entire tool-kit involves multiple modalities, and represents the goals of individuals and collectives but, unlike language, it is not arbitrary and compositional, and is functionally limited to the here-and-now of the communication event. It allows for explicit cooperation at all the relevant levels, from information exchange, through explicit teaching of manual skills (in tool-making, hunting etc.), all the way to the maintenance of social life (through both micro-interactions and collective rituals). The implications of mimetic communication for the vocal modality in particular are far-reaching. Better executive control would have improved vocal learning abilities in humans, increasing the repertoire of vocalizations and making their use more flexible.

An additional factor affecting vocal flexibility is the relaxation of selection. Studies comparing the birdsong of white rumped munia to that of its domesticated strain, the Bengalese finch, show that relaxed selective conditions enable vocal learning that is less constrained than that observed in the wild and eventually leads to more complex songs (Okanoya, 2015). This implies that, in addition to the benefits of improved executive control, extended juvenile periods and more buffered human habitats may have also increased the variability and complexity of human vocal communication. A flexible and extensive use of vocal communication in the lives of hominins would have set the stage for the elaboration of the vocal modality in musical engagement and language.

We agree with Donald that mimetic communication and mimetic cognition are sufficient to account for the undoubtedly rich, yet in other senses limited, Acheulian cultural complex (Shilton, 2017). Although the skills and knowledge required for producing Acheulian stone tools and hunting megafauna are impressive, their social transmission is dependent mostly on cooperative interactions in the here-and-now, and do not require the extended functionality of language (described in the following section). As previously mentioned, the social transmission of both tool-making and foraging skills requires that skilled individuals share with novices their way of looking at and responding to the environment. Recognizing visual cues is essential for skills such as finding suitable raw materials for tools, identifying a good striking platform on a core, spotting the spoors of prey and predators, and locating underground storage organs. Mimetic communication would have enabled hominins to coordinate the way they perceived and engaged with the environment they experienced together – to reduce what Dor (2015) calls “experiential gaps,” the inescapable differences in the way different individuals experience their surroundings. Mimetic communication, along with social motivation and theory of mind, can enable ensuing processes of what Dor calls “experiential mutual identification,” in which hominins direct the attention of their counterparts to elements of interest in their immediate environment, attempt to share their attitudes toward them, and construct a mutually-identified collective view of the environment. This results in the creation of an intersubjective common ground, enabling flexible coordination within the here-and-now. By continually engaging in experiential mutual identification, hominins could transmit the diverse knowledge and skills they were continually acquiring. Hominin codependence would create a new evolutionary spiral in which new cooperative behaviors would continuously require upgrades to the toolkit of mimetic communication, the upgrades would enable new cooperative behaviors, which would increase codependence, and so on – an ever extending spiral of positive feedbacks, one in which humans may be said to be caught up in to this very day.

We believe that musicking played a crucial role in this process. Much has been written on the importance of music in human evolution, and we can address this literature only briefly (for a more thorough discussion, see Cross, 2007). It was discussed by Darwin (1871), who suggested musical behavior, grounded in the vocal expression of emotions and operating in the context of mating and sexual selection, was a precursor of language. After several decades of relative silence on the subject, interest revived in the 1990s, and was reinvigorated by Pinker’s (1997) provocative and arguably ethnocentric claim that music is an “auditory cheesecake.” This claim, which was based mainly on Western habits of passive music consumption, ignored the fact that in most of human history and for most human cultures musicking was and remains a participatory and highly social activity. Mithen (2006), who contributed substantially to the discussion, described musicality as part of the mimetic toolkit and envisioned a role for it in prehistoric lives.

We agree that musicking is mimetic in essence, but also think that some of its unique qualities merit a separate discussion and special recognition. One such quality is its anticipatory nature. Music contains tonal and rhythmic elements which are meant to trigger an embodied anticipation of its continuation. This anticipation relies mainly on rhythmic entrainment and repetition. Rhythmic entrainment, or beat-based timing, differs from interval-based timing (which has been documented for some primates) in that movements anticipate the onset of the musical beat, rather than merely corresponding roughly to the musical beat period (Merchant and Honing, 2014). Repetition is a universal quality of music (Nettl, 1983) and can even endow speech and random tone sequences with a perceived sense of musicality (Deutsch et al., 2011; Margulis and Simchy-Gross, 2016). Most importantly, repetition triggers more forcibly the anticipation of the next beat or sound. By supplying an anticipatory tonal and rhythmic foundation for play interactions and group mimetic acts, musicking substantially extends the potential for creating emotional synchrony and rituals of social bonding. Musicking is different from other forms of mimetic communication (as well as from language) because it establishes simultaneous rather than asynchronous interactions (Cross, 2016), as well as carrying highly embodied and ambivalent meanings (Langer, 1957; Cross and Tolbert, 2016). Musicking, unlike language, enables big groups to express themselves together; and while language excels at displacement, musicking is unusually potent in synchronizing the embodied experiences of participants, and with it, their arousal and emotional states.

We consequently suggest that musicking is a technology of engagement: communicative messages that are designed to strongly compel the receiver to emulate their rhythm and tonality. Music perception reflects this anticipatory nature of musicking by being highly embodied, predictive and, in a sense, inherently active. Beat perception, for example, is defined by the ability to predict the next beat, and engages motor areas of the brain regardless of any overt movement (Patel and Iversen, 2014). Listening to melodies similarly involves making involuntary predictions about their continuation (Margulis, 2005). Since there are non-arbitrary relationships between tempo, pitch, timbre and certain emotional states (Juslin and Laukka, 2003), and since emotional contagion based on automatic bodily mimicry results in emotional convergence (Hatfield et al., 1994), musical synchrony necessarily translates into emotional synchrony. This makes musical engagement a potent tool for emotionally uniting humans and for enhancing group cohesion and trust, which is particularly important during cooperative activities like hunting big animals or fighting with rival groups. As Darwin (1871) noted, social cohesion and solidarity would have a strong selective value at the group level.

While several species are capable of rhythmic entrainment, so far only parrots have been shown to respond to music spontaneously and with diverse movements (Keehn et al., 2019). This has led Keehn et al. (2019) to suggest five traits that are necessary for rhythmic entrainment: complex vocal learning, a capacity for imitation, an ability to learn complex action sequences, a tendency to form social bonds, and attentiveness to communicative movements. Wilson and Cook (2016) argue that what distinguishes parrots from other animals are two critical factors: social motivation and voluntary motor control. If so, it suggests that selection for executive control and pro-sociality would have made hominins responsive to rhythmic stimuli. But whereas parrots spontaneously respond to music with diverse movements, they do not *make* music. For hominins to create and develop this new form of communication, two other abilities were needed. First, proficiency in mimetic communication, which enables the flexible and intentional production of iconic bodily signals in a cooperative context; and second, the ability to create and sustain cumulative cultures, thus forming increasingly complex traditions of rhythmic and tonal group engagement.

Although it is difficult to establish whether musical engagement was directly or indirectly selected when it first appeared, it seems that the ability to engage in musical interactions is strongly related to other traits that are likely to provide fitness benefits, such as improved vocal and motor control, pro-social motivation, as well as good social skills and empathy (Keller et al., 2014; Novembre et al., 2019). Musical engagement could initially have evolved as a particularly engaging form of play and social grooming that was based on synchronous tapping, vocalizations and movements. Based on the ethnography of contemporary African hunter-gatherers, Lewis (personal communication) suggests that the first critical role of musical engagement was in deterring nocturnal predators. In time, musical engagement began to play a significant role in many other aspects of social life. Music’s unique properties make it the only form of communication which allows several individuals to express themselves simultaneously as a single group, thus contributing substantially to social bonding, acculturation and the creation of group identity (Lewis, 2016). These contributions were probably adaptive at both the group level (more cohesive groups were more successful than less cohesive ones) and at the individual level (individuals who participate in musicking were trusted more than those who did not).

Studies on the neurochemistry of music point to its influence on factors related to reducing stress and enhancing social bonding (Chanda and Levitin, 2013). A meta-analysis of music therapy studies concluded that it is effective in reducing pain and anxiety (Kühlmann et al., 2018), something which appears to be related to reducing levels of cortisol and ACTH (adrenocorticotropic hormone). Listening to soothing music was found to increase oxytocin levels during post-surgery bed rest (Nilsson, 2009), and Kreutz (2014) found that, compared to dyadic chatting, group singing increased oxytocin levels, as well as significantly enhancing perceived psychological well-being. The pleasure derived from listening to music appears to be modulated by dopaminergic reward systems (Ferreri et al., 2019), and a PET study documented dopamine release in striatal regions during both peak arousal and in anticipation of it (Salimpoor et al., 2011). Tarr et al. (2014) also mention the likely influence of musical engagement on the endogenous opioid system, with exertion-related release of endorphins during musicking promoting social bonding.

Whatever the neurochemical mechanism, the influence of music on social bonding is well documented. Several studies have shown that movement synchrony alone promotes pro-sociality (Cirelli, 2018), with some finding positive effects on peer cooperation (e.g. Rabinowitch and Meltzoff, 2017). Reviewing the interpersonal effects of movement synchrony, Cross et al. (2019) highlight deindividuation, where the sense of self is diluted and one comes to feel less separate from others. The general effects of movement synchrony therefore provide the basis for collective emotions, which are intensified by the wide range of feelings embodied and induced through musical engagement, and by the creation of musical traditions unique to specific social groups.

Because musical interactions are based on the synchrony of embodied experiences, they can be a powerful tool for uniting a group in a single, socially mandated “mood,” be it calm, joy, grief, anger (directed at another group) or ecstasy. Musicking continued to diversify alongside the emergence of language and more complex social structures, being utilized for a variety of social functions. Cross-culturally, musical engagement appears in broadly similar social practices, notably dance, ritual, religious ceremonies, processions, mourning, healing and infant care (Mehr et al., 2019). These diverse utilizations of musical communication in contexts that are critical for harmonious social life confirm its importance as a tool for modulating the emotions involved in collective activities and in responding to social demands. Musical engagement, made possible by a selection for emotional and executive control and pro-sociality, became a potent tool for promoting further pro-sociality, improving executive control, and inducing socially prescribed emotional states.

### Language and Emotional Control

Mimesis could maintain the various cooperative behaviors we have described to a level that was probably sufficient for more than a million and half years. However, as codependency in hominin groups increased, it gradually required a system of communication that could break the boundaries of the here-and-now of the communication event, and allow the communication of experiences, norms, skills and worldviews beyond what was possible through mimesis. The new system of communication was language. It was built on the basis of mimesis, with the first prototypes of language appearing around a half a million years ago (Dediu and Levinson, 2013), and continued to evolve in a process of culturally driven, gene-culture coevolution until it acquired its fully fledged form (Dor and Jablonka, 2014).

All the tools of communication that we share with our ape relatives, and the toolkit of mimesis that is uniquely human, share a basic functional strategy: they enable communicators to target their interlocutors’ senses, and present them with communicative materials to perceive. As Dor (2015) shows, the functional uniqueness of language lies in the fact that language abandons this strategy – it allows speakers to communicate directly with their interlocutors’ imaginations. It permits speakers to intentionally and systematically instruct their interlocutors in the process of imagining the intended meaning instead of experiencing it. Speakers provide interlocutors with a code, a structured list of the basic co-ordinates of their experience, which the interlocutors then use as a scaffold for their own imagination. Following the code, the interlocutors raise past experiences from their own memories, and then reconstruct and recombine them to produce novel, imagined experiences. Language is thus the only system that allows the communication of meanings that cannot be presented to the senses. This includes experiences from the past and from other places (this is Hockett’s displacement: reference to things remote or “displaced” in time and space), but also, and as importantly, a very wide variety of inner experiences that are very difficult to present, even if they refer to the here-and-now. The fact that the communicator is worried, for example, may show itself on his or her face, but if the object of worry is not directly available for perception, it will remain uncommunicable without language. Language makes it communicable, and it does so on the basis of the collective effort of experiential mutual-identification that has already been established in the mimetic period. The crucial upgrade is that every point of experiential mutual-identification is symbolically marked by a mutually identified sign – lexical, morphological or syntactic. This symbolic signification allows speakers to translate what they want to communicate into formally arranged symbolic codes and transmit the codes to their interlocutors. The interlocutors analyze the codes, retrieve from their memories the relevant experiences that are associated with the signs, and construct their own imagined experiences.

Language thus revolutionized hominin life. For the first time, individuals could begin to take into account things they themselves have never experienced, things they only heard about. Communities could begin to explicitly negotiate collective conceptualizations of the world, norms of social conduct, and plans for future collaborative activities, all of which in the mimetic period could only be implicitly and indirectly negotiated through perceptible behavior (Dor, 2019). Stories (both factual and fictional) became a crucial mode of information transfer, identity synchronization and negotiation of social behavior and norms (Smith et al., 2017; Boyd, 2018), and conversations allowed explicit complaints and criticism (Wiessner, 2014).

As we see it, once in place, the evolution of language must have entailed profound alterations to hominins’ emotional profiles and in their capacities for emotional control. At the most foundational level, the emergence of a linguistic communication technology that transcends individuals’ immediate experiences of the here-and-now required them to develop increasing levels of trust and the control of affect-related drives and triggers of action. When told about things beyond what they could perceive by themselves, whether dangerous or beneficial to them, individuals had to imagine those things while either inhibiting, modulating or mobilizing the appropriate emotional response. They also had to face new problems: they needed to reduce the dangers of false memories and distinguish between what they recalled on the basis of their own experiences and what they recalled on the basis of stories told by others. These dynamics led, among other things, to the evolution of the human-specific phenomenon of distinguishing between thought and feeling (Jablonka et al., 2012).

The instruction of imagination, which is what language enables, has another problematic aspect: it revolutionized deception and enabled the uniquely human phenomenon of the lie. Many evolutionary-oriented scholars argue that this new capacity for lying was a major obstacle to the emergence and stabilization of language itself (Boyd et al., 2003; Knight, 2007; Mercier and Sperber, 2011; Tomasello, 2016). Their argument is based on the idea that linguistic communication requires trust: if everybody lies to everybody else, the trust breaks down, and language itself follows suit. As Dor (2017) shows, however, this line of reasoning is based on a series of unrealistic assumptions. It concentrates on a single type of lying, where an individual lies with an explicit exploitative intention, and the lying carries real detrimental consequences for the community, but this is far from representing the functions of lying in linguistic communication. Individuals very often lie with non-exploitative intentions, sometimes with pro-social intentions (‘white lies’), and such lying actually contributes to social cohesion. Potentially detrimental and exploitative lies are effectively policed and punished in small groups, so exploitative lying is unlikely to destroy linguistic communication. Moreover, language is not restricted to the transfer of propositional information. It is necessary for collective action and collective identity, so the multiple functions of language compensated for its occasional and inevitable detrimental effects. Especially relevant to our current discussion is the fact that lying, both exploitative and non-exploitative, requires more sophisticated capacities at the cognitive, emotional and social levels, than honest communication. It did not harm the overall capacity of the community to cooperate, but made the social negotiation of community life more nuanced and broader in scope. Lying requires higher levels of emotional control of behavioral expressions – bodily, facial and vocal – than honest communication (Dor, 2017); efficient lying requires a poker face and the ability to express pretended emotions. Language, therefore, would have added to the selective pressures for better mimesis-related emotional control, rather than reducing the need for it. A similar dynamic would have occurred at the underlying physio-anatomical level of adapting to language, as the appearance of the modern vocal apparatus made the human face highly mobile and controllable, thus increasing the repertoire of facial expressions and their voluntary control (Donald, 1991; Wilkins, 2017). In addition, language was arguably able to provide its own means for emotional control, whether for lying or other purposes. Neuroimaging studies show that stimulus reappraisal, a widely acknowledged cognitive process of emotion regulation (Ertl et al., 2013), is correlated with activity in brain areas that are involved with the representation of semantic knowledge and its retrieval (Wagner et al., 2001; Satpute et al., 2014). Although semantic knowledge is not necessarily linguistic, its digitization and massive expansion during the emergence and development of language (Dor, 2015) could have allowed hominins living in linguistic groups to better categorize, appraise and reappraise emotion-provoking stimuli, and thus better control their responses to them.

In addition, the mutual identification, categorization and signification of emotional experiences led to the emergence of a semantic field of emotion – sets of semantically related words and expressions referring to mutually identified emotions. Emotion-words enable affect labeling, a language-specific technique of emotion regulation, which can modulate an emotional experience, its accompanying physiological response and the resulting behavior, in accordance with the emotion-word used for categorizing the initial affective response. For example, a stress response can be categorized by an emotion-word as either exciting or fearful, and this alters the resulting emotional experience, the physiological correlates and the behavioral responses of the individual that utters or responds to the emotion-word (Jamieson et al., 2013). Another possible contribution of emotion-words for emotional control is in their use as scaffolds for endogenous emotion generation, a process which in itself can be used to regulate emotional responses to external stimuli (Engen and Singer, 2018). For example, an emotion word such as “anger,” when used in the context of a conflict with an out-group member, could help mobilize an aggressive response.

Hare (2017) cites evidence that the widening of the developmental window enables human children to reach, around age 6, levels of self-control that exceed those of non-human apes. It is around this same age that children begin to internalize means of emotion regulation including speech signs, so audible taunts and curses become silent ones, a visible smile becomes an inner smile, and on the linguistic level, audible speech becomes inner speech (Holodynski and Friedlmeier, 2006). Symbolic strategies are increasingly employed by caregivers to instruct the children under their care, teaching the children why and how they should control and express their emotions (Holodynski and Friedlmeier, 2006). Such a dynamic puts the individual child’s unique emotional profile and its expression under collective pressure, making him comply with shared cultural norms and reflect on his emotional state and its regulation.

The emergence of language in hominin evolution added to shared cultural norms a gradually increasing subset of language-specific norms of communication, such as conventionalized conversational styles. This would have placed additional selective pressures on individuals’ capacities for emotional control. Living among egalitarian, coordinated groups requires a heightened sensitivity to the motives and emotional states of others, especially while negotiating smooth interactions between group members. As data from modern hunter-gatherer societies show, these requirements may result in pervasive conversational styles of surface courtesy, which are achieved through a conspicuous and conventionalized politeness (Brown, 2004; Groark, 2008). The effects of these or other norms of linguistic communication on the emotional lives of their speakers may vary according to how different linguistic groups (and different group members within them) view the relations between language and experience (Dor, 2015). For example, in Tenejapa Mayans, conspicuous politeness, which includes politeness utterances, serves to convey agreement, empathy, and positive affect (Brown, 2004), thereby promoting pro-sociality. In Tzotzil Mayans, on the other hand, aggression has been transferred from the physical level (assaults and murders are relatively uncommon) to the symbolic-linguistic level. Ill-wishing utterances are believed to possess a sorcerous quality when uttered within the privacy of one’s residency, often during the night, i.e. away from everyday social interaction and its linguistic norms of politeness (Groark, 2008).^3^

Language has also led to the cultural construction of novel categories of feelings (or emotions; we use the terms interchangeably here). For example, feelings of certainty, suspicion and doubt derive from issues of truth and falsity as properties of the relationship between a linguistic message – arbitrary and displaced – and the experiential world (Jablonka et al., 2012). Other existing, prelinguistic feelings came to be mutually identified and reconceptualized in ways that align with the values, myths and the shared worldview of a linguistic group’s semantic landscape. As Myers (1988) shows in his conceptual analysis of ‘compassion’ and ‘anger’ in the culture and language of Pintupi Aborigines, these emotion-words refer to the acceptance or rejection of relatedness. “Compassion” refers to the acceptance of relatedness and “anger” to its rejection. Generally, once people construct overall shared worldviews through language and myth, their linguistic emotion-concepts will come to reflect this “deep structure.”

At the simplest level, the sharing of experiences through language has allowed individuals to expand their private experiential knowledge, including emotional knowledge (Jablonka and Ginsburg, 2012). This sharing of emotional knowledge could have been achieved by the use of metonyms and metaphors (e.g. the “head” of the group; having the upper “hand”), which derive from the shared anatomy and physiological functioning of the human body and appear to be a universal tool for cultural-specific content (Kövecses, 2000). Linguistically constructed emotion concepts that are combined together further elaborate the semantic emotional knowledge of individuals (Barrett, 2017). They bring together diverse bodily sensations, thoughts, feelings, and social contexts in unique configurations.

## Conclusion and Future Directions

Domestication is the longest and the most systematic evolutionary experiment that humans have ever conducted. It was used by Darwin (1872) to explain evolutionary change through natural selection and assortative mating, and in the *Variation in Animals and Plants under Domestication* (Darwin, 1868) to shed light on the generation of heritable variations. It has profoundly changed the history of humans, being a necessary condition for the agricultural revolution (Diamond, 1997), and is used today as an example of evolutionary change that highlights the need to incorporate multiple modes of information transmission (genetic, epigenetic, behavioral and cultural) when considering cumulative evolution (Zeder, 2018). The social evolution of humans and bonobos has been interpreted as a special variant of domestication – as a self-domestication process. While this analogy has led to productive research because it focused attention on the commonalities of humans and domesticates, we believe that the social evolution of humans is better explained in terms of selection for pro-social motivation and self-control, which are guided by symbolic communication and representation rather than as a process of self-domestication.

In this paper we have emphasized the differences between the evolution of domesticates and the social evolution of humans. First, while in domesticates there is a breakdown of social structures (Spurway, 1955), social structures in humans became more complex. Second, in most domesticates there is a reduction in brain size whereas brain size increased during most of human evolution. It is possible that in humans the last 10,000 years of sedentary life led to small-island-like conditions, which could arguably explain the recent reduction in human brain size (Sánchez-Villagra et al., 2016). Generally, however, although selection for reduced emotional reactivity was involved in both socially impoverished domesticates and socially sophisticated humans, the differences between the two types of pro-social evolution mean that their inclusion under the same umbrella term of “domestication” is misleading.

Third, the evolution of all cooperative and sophisticated social animals, including humans, was inevitably entwined with changes in their emotional dispositions. We observe, on the one hand, context-sensitive reduced aggression and displays of affection toward some group members. Notable examples are teaching the young by non-parents in meerkats, alloparenting in wolves, and sophisticated, hierarchical social structure and reduced aggression of mole rats (Skulachev et al., 2017). On the other hand, in some of the same cooperative species we find increased aggression toward group members, mainly in the context of status-related conflicts. For example, the offspring of a subordinate meerkat females that have become pregnant are killed by the dominant female, and such subordinates are often evicted from the group; in addition, subordinates may engage in infanticide activities, though to a significantly lesser degree (Clutton-Brock et al., 1998). Proactive violence against other groups is also evident in meerkats and other cooperative species. What is striking about these and most other examples of social behavior in cooperative taxa is the increased context-sensitivity of both pro-social and aggressive behaviors, which points to an altered emotional responsiveness. We therefore expect that comparing humans with other social mammals will be as fruitful as comparing them to domesticates. We anticipate that future research will uncover similarities in the executive control of emotions among humans and other highly social mammals, which will only partially overlap the early developmental pathways that are affected in the DS. It would be of particular interest to study the correlations between neoteny, increase in brain size and alloparenting practices in humans and other highly social mammals, and compare the developmental networks that underlie these cooperative behaviors.

More specifically, we expect that the developmental pathways and the genetic and epigenetic networks underlying cooperative behavior, neoteny and other features related to pro-sociality in humans and other social mammals will include neural crest-related gene networks. These networks underpin cranial differences and other important morphological and physiological changes that are involved in domestication and have been targeted by selection during the social evolution of humans. However, we predict that in humans, changes in the GRNs underlying the HPA axis and pathways associated with learning and with the control of emotions, will be even more prominent. The nature of the changes in the cognition and emotionality of humans suggests that pathways controlling metacognition (e.g. complex decision-making and regulation of affect), which are controlled by neo-cortical regions, were important targets of selection. These pathways are also expected to underlie the social evolution of other mammals that became socially organized and cooperative with regard to tasks such as foraging, hunting, group defense and alloparenting. Hence we predict that genetic and epigenetic changes in the GRNs underlying the development of these pathways will prove to be of major importance in the evolution of highly social mammals and to be especially prominent in human evolution.

Extending Hare’s (2017) suggestion, we have argued that the evolution of emotional self-control is the hallmark of human social evolution, and that self-control is part of the cognitive-affective evolved make-up of humans. We identified two overlapping stages in the process, one preceding the emergence of language and the second following it. The first stage involved the initial development of the hominin life-style: hunting and foraging, toolmaking, and alloparenting. Each of these practices both profited from and promoted an increase in emotional control and pro-sociality. As argued elsewhere (Dor and Jablonka, 2010, 2014; Dor, 2015), the evolution of all forms of uniquely human communication – including mimesis, musical engagement and language – was driven by cultural evolution. (For extensive discussion of human cultural evolution see Richerson and Boyd, 2005; Mesoudi, 2011; Jablonka and Lamb, 2014; Henrich, 2016; Laland, 2017.) The evolution of human communication was initiated by cultural practices that shaped the plastic human brain through learning-based adjustments, followed by the partial genetic accommodation of elements enabling ever more complex communication. Hence, culturally evolved communication not only adapted to the brains and minds of individual communicators, but the brains and minds of the communicators became adapted to the culturally evolving communication systems, thereby generating, through positive feedbacks, an ever-widening co-evolutionary spiral. We have argued that the evolution of pro-sociality and cooperation in pre-linguistic humans was a major part of this spiraling evolutionary process. Among other things, it entailed the evolution of increased emotional control and mimetic communication, including rhythmically entrained mimetic acts that are the seed of musical engagement. Building on the foundation of rhythmic and tonal anticipation, musical activities became a powerful technology of engagement, capable of inducing high levels of emotional synchrony and promoting pro-social behavior. Because of this, we expect to find that limbic regions involved in emotional reactions became specialized during human evolution, as were the neo-cortical regions controlling them (for some indications that this may indeed be the case, see Barger et al., 2014.) According to our argument, the basic social emotions of embarrassment, shame, guilt and pride evolved in this context. We expect that once the developmental genetic networks underlying them (and the blush, which is their outward expression) are identified, the relevant variations will be found to be shared by modern humans and their Neanderthal and Denisvoan cousins.

The second stage that we identified in human evolution involved a further increase in emotional and cognitive plasticity that was driven by the instruction of imagination through language. We therefore expect, and find, a strong cultural influence on the expression of emotions, including the ability to suppress emotions under some social and intellectual conditions. We also expect, and find, that the new adaptations in the ability of humans to represent and to communicate have led to a huge increase in the ability to deceive and to the problem of distinguishing between false and true memories, problems that were partially solved by cultural evolution of social norms and the development of autobiographical memory (Jablonka, 2017; Dor, 2019). More culture-specific changes in the control and effects of emotions can also be explained within this framework. Sapolsky (1999), for example, discusses the case of individuals with repressive personalities whose “unemotional” lives of discipline and conformity are actually characterized by markedly elevated basal cortisol levels and hyper-reactive sympathetic stress-responses. These findings are notable because repressive personalities are not exposed to irregular amounts of stress with which they are coping maladaptively, but rather are highly stressed by the process of constructing a world without any stressors. We see this case as illustrative of the ways in which human-specific emotional control and emotional plasticity, mediated by language and other uniquely human cultural practices, may modulate and mold human physiology, and in doing so lead to a phenotype that partially resembles that of domesticates.

In summary, we regard the social, gene-cultural evolution of humans as more similar to the social evolution of other highly social mammals that display enhanced cognitive and affective plasticity and sophisticated social structures, than to the evolution of socially impoverished domesticates. These similarities however, pale in comparison to the unique features of human social evolution, which has been guided by cumulative cultural changes that led to increased cognitive and affective plasticity, allowing feats of saintly cooperation and sadistic cruelty that go far beyond those of any other animal.

## Author Contributions

All authors listed have made a substantial, direct and intellectual contribution to the work, and approved it for publication.

## Conflict of Interest

The authors declare that the research was conducted in the absence of any commercial or financial relationships that could be construed as a potential conflict of interest.

## Footnotes

1 The term “domestication syndrome” (DS) was first used to describe the suite of characters shared by animal domesticates by Wilkins et al. (2014).

2 The observation that modern humans and wolves do not share variations in recently selected human genes (Theofanopoulou et al., 2017) is not surprising given the recent origin of these genes in humans and the far more ancient origin of wolves, which seem to have diverged from the highly social and cooperative coyotes 1.5 million years ago and apparently have not undergone intense social evolution since then.

3 The symbolic norms regulating human behavior, which are culturally learned and culturally evolved, make it possible to argue that humans are “domesticated” by externally imposed symbolic social conventions (and by an internally constructed, norm-binding “super-ego”). This notion of HSD, which goes back to the old and misleading idea of humans being tamed by “civilization,” is very different from the current one. Moreover, the regulation of human life by symbolic norms and conventions has little in common with the taming of domesticates, or with the social evolution of bonobos and other social mammals.
